# Protective measures and H5N1-seroprevalence among personnel tasked with bird collection during an outbreak of avian influenza A/H5N1 in wild birds, Ruegen, Germany, 2006

**DOI:** 10.1186/1471-2334-9-170

**Published:** 2009-10-18

**Authors:** Wei Cai, Brunhilde Schweiger, Udo Buchholz, Silke Buda, Martina Littmann, Jörg Heusler, Walter Haas

**Affiliations:** 1 Department for Infectious Disease Epidemiology, Robert Koch Institute, DGZ Ring 1, 13086 Berlin, Germany; 2 German National Reference Centre for Influenza, Robert Koch Institute, Nordufer 20, 13353 Berlin, Germany; 3 State Office of Health and Social Affairs Mecklenburg-Western Pomerania, Gertrudenstrasse 11, 18057 Rostock, Germany; 4 Health Office Ruegen, Gartenstrasse 1, 18528 Bergen, Germany

## Abstract

**Background:**

In Germany, the first outbreak of highly pathogenic avian influenza A/H5N1 occurred among wild birds on the island of Ruegen between February and April 2006. The aim of this study was to investigate the use of recommended protective measures and to measure H5N1-seroprevalence among personnel tasked with bird collection.

**Methods:**

Inclusion criteria of our study were participation in collecting wild birds on Ruegen between February and March 2006. Study participants were asked to complete a questionnaire, and to provide blood samples. For evaluation of the use of protective measures, we developed a personal protective equipment (PPE)-score ranging between 0 and 9, where 9 corresponds to a consistent and complete use of PPE. Sera were tested by plaque neutralization (PN) and microneutralization (MN) assays. Reactive sera were reanalysed in the World Health Organization-Collaborating Centre (WHO-CC) using MN assay.

**Results:**

Of the eligible personnel, consisting of firemen, government workers and veterinarians, 61% (97/154) participated in the study. Of those, 13% reported having always worn all PPE-devices during bird collection (PPE-score: 9). Adherence differed between firemen (mean PPE-score: 6.6) and government workers (mean PPE-score: 4.5; p = 0.006). The proportion of personnel always adherent to wearing PPE was lowest for masks (19%). Of the participants, 18% had received seasonal influenza vaccination prior to the outbreak. There were no reports of influenza-like illness. Five sera initially H5-reactive by PN assay were negative by WHO-CC confirmatory testing.

**Conclusion:**

Gaps and variability in adherence demonstrate the risk of exposure to avian influenza under conditions of wild bird collection, and justify serological testing and regular training of task personnel.

## Background

Severe human A/H5N1 infections were first observed during outbreaks of highly pathogenic avian influenza (HPAI) A/H5N1 among poultry in Hong Kong in 1997 [1]. Since its re-emergence in Asia in 2003, 438 human cases have been reported worldwide, of which 60% had a fatal outcome (as of 11 August 2009) [2]. The main risk factor for human A/H5N1 infection is direct contact with HPAI A/H5N1-infected animals [3].

In Germany, the first outbreak of HPAI A/H5N1 occurred among wild birds on the island of Ruegen in the federal state Mecklenburg-Western Pomerania, in northeastern Germany between 8^th ^February and 6^th ^April 2006. Of 1,881 tested birds, 8.4% were laboratory confirmed H5-positive. The most commonly affected birds were wild swans (90%) [4]. Soldiers of the German Federal Defence Force, professional firemen from Mecklenburg-Western Pomerania, firemen of the local auxiliary fire brigade and local government workers (administrative staff) participated in collection of wild birds on Ruegen during the outbreak. In addition, local veterinarians collected and transported wild birds to laboratory for testing.

**Figure 1:**
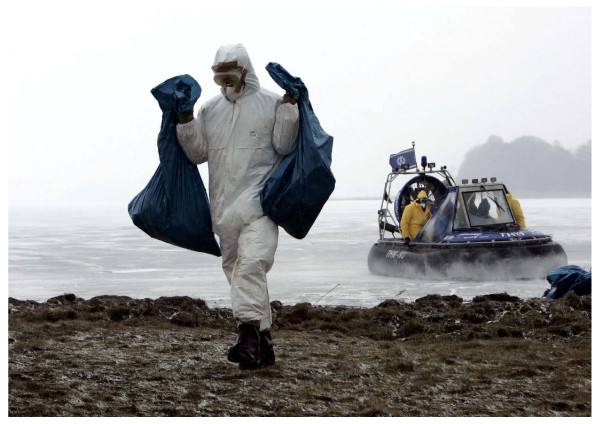
**Conditions and PPE use of personnel tasked with bird collection during an outbreak of avian influenza A/H5N1 in wild birds, Ruegen, Germany, 2006**. Source: Jens Koehler/ddp.

The Ruegen Health Office recommended protective measures for personnel tasked with wild bird collection according to official German recommendations [5-7]. These included use of personal protective equipment (PPE: headwear, protective goggles, masks, protective clothing, gloves and protective boots; Figure 1) during bird collection and current seasonal influenza vaccination. Acting on the recommendation of the State Office of Health and Social Affairs Mecklenburg-Western Pomerania, antiviral prophylaxis (oseltamivir) was not recommended for the personnel tasked with bird collection who collected the birds using recommended PPE.

So far, no human A/H5N1 infection has been reported in Germany. However, the possibility of asymptomatic A/H5N1 infection after exposure to potentially A/H5N1-infected poultry has been reported in different countries and the prevalence can be as high as 3-10% [8,9].

Poultry is known to have played a major role in the epizootic transmission of avian influenza to humans [10]. To date, only limited studies have been carried out to investigate the transmission of avian influenza to humans by close contact with potentially infected wild birds during outbreaks [11,12].

We launched an investigation to assess adherence to the use of protective measures among personnel tasked with wild bird collection during the HPAI A/H5N1 outbreak on Ruegen in order to improve recommendations to prevent exposure by potentially HPAI A/H5N1-infected animals. In addition, we searched for clinical symptoms of personnel enrolled during and after wild bird collection and measured their seroprevalence of anti-H5N1 antibodies in order to assess the risk of human A/H5N1 infection.

## Methods

### Study participants

We organized several information sessions on Ruegen in March 2007 in order to motivate study participation. Inclusion criteria of our study were participation in collecting wild birds on the island of Ruegen between February and March 2006. As soldiers of the German Federal Defence Force and professional firemen (according to the Ruegen Health Office: about 400 soldiers and 34 professional firemen) had been recruited for bird collection from all parts of Germany and could not be contacted at the time of investigation, they were not included in this study.

### Ethical clearance and data protection

The study was approved by the Ethics Commission of the Charité, Universitätsmedizin, Berlin and the Commissioner for Data Protection and Freedom of Information of the German Federal Government and the State of Mecklenburg-Western Pomerania.

### Data collection

Persons who agreed to participate in this study were asked to give a written informed consent and to complete a questionnaire soliciting demographics (date of birth, sex, occupation), conditions of wild bird collection (species and status of collected birds, finding situation), use of protective measures during bird collection (kinds of used PPE-devices and frequency of PPE use), problems regarding PPE use (difficulties in adherence to PPE use, risk behaviour that could reduce the protective effect of PPE), seasonal influenza vaccination status, and acute respiratory symptoms during and up to 5 days after bird collection [see Additional files 1 and 2].

Influenza-like illness was defined as the presence of fever, cough, headache and muscle or limb pain.

### Evaluation of PPE use

To evaluate adherence to the use of protective measures we constructed a PPE-score. The score integrated both completeness and frequency of use. Generally, masks, protective clothing, gloves and protective goggles were reported to be most effective PPE-devices against influenza virus [13,14]. However, we considered goggles less effective against A/H5N1 infection as in contrast to other avian influenza subtypes (e.g. A/H7) conjunctivitis was rarely reported as clinic manifestation of A/H5N1 infection [3]. Therefore, we considered masks, protective clothing and gloves more effective to protect personnel tasked with bird collection than headwear, protective goggles and protective boots, and these were assigned scores of 2 and 1, respectively, if they were "always" used during bird collection. When a PPE-device was "sometimes" used, half the score was assigned (Table 1). Therefore, a person who indicated that he or she "always" used all PPE-devices during bird collection obtained a maximal score of 9 (3*2 + 3*1). We also calculated the PPE-device specific "adherence ratio" as the sum of all scores obtained for a specific PPE-device by all participants divided by the maximum possible score multiplied by the number of participants.

**Table 1:** Scoring system for use of PPE according to frequency of use

	Yes, always	Sometimes	No/Don't know/No response
**Masks**	2	1	0
**Protective clothing**	2	1	0
**Gloves**	2	1	0
**Headwear**	1	0.5	0
**Protective goggles**	1	0.5	0
**Protective boots**	1	0.5	0

### Serological testing

Persons who agreed to participate in this study were asked to provide a single 5 mL blood sample, which was sent to the German National Reference Centre for Influenza (GNRCI), Berlin. Unrefrigerated transport to GNRCI took a maximum of 24 hours. Serum was extracted from blood and stored at -20°C at GNRCI until tested for antibodies against A/H5N1 virus.

The sera were tested by plaque neutralization (PN) and microneutralization (MN) assays using the reference virus strain A/whooper swan/R65-2/Germany/2006 (H5N1), which was directly taken from an infected swan during the outbreak on Ruegen. Reactive sera were reanalysed by the World Health Organization-Collaborating Centre for Reference and Research on Influenza (WHO-CC), London by MN assay using the reference virus strains A/bar-headed goose/Qinghai/1A/2005 (H5N1) and A/whooper swan/Mongolia/244/2005 (H5N1), which were isolated from infected birds in Asia. The reference strains used by GNRCI and WHO-CC belong to a same cluster of clade 2.2. Serum samples were considered to be reactive by PN or MN assay if anti-H5 titre was > 1:20. In addition, MN assay was performed by GNRCI using reference virus strains A/New Caledonia/20/99 (H1N1) and A/Wisconsin/67/05 (H3N2) to analyse possible presence of cross-reactive antibodies to human influenza A/H1N1 and A/H3N2.

### Statistical analysis

The data were analysed using Excel (version 11, Microsoft Corporation, Redmond, Washington, USA) and Stata (version 9.0, StataCorp LP, College Station, Texas, USA). Mean PPE-scores were presented with a 95%-confidence interval (95%-CI). Associations between human influenza vaccination status and PN assay result and between human influenza vaccination status and acquiring acute respiratory symptoms were tested by chi square test. Fisher's exact test was used when expected values for cells below 5. The Mann-Whitney test was used to assess influencing factors to PPE- score. A p-value of less than 0.05 was considered statistically significant.

## Results

### Study participants

Of the eligible 159 (96% male) firemen of the local auxiliary fire brigade, local government workers and veterinarians, 97 (61%) participated in this study. All study participants were residents of Ruegen. Within the subgroups, government workers had the highest participation rate (88%; 21/24), ahead of veterinarians (83%; 5/6) and firemen (55%; 71/129). Of the 97 participants, 94 completed the questionnaire, of whom 90 (96%) were male. The median age of participants was 36 years (range: 18-60 years).

### Conditions of wild bird collection

According to the reports given by participants, the most common species of collected birds were wild swans (86%). Among the participants, 82% (58/71) reported having collected wild birds from water or ice surfaces, 71% (52/73) reported having collected frozen birds, 88% (64/73) wet birds, and 47% (40/86) reported having collected live birds.

### PPE use during bird collecting

Of 94 participants, 12 (13%) reported having always worn all PPE-devices during bird collection (PPE-score: 9); 91 (97%) reported having ever used at least one PPE-device during bird collection. The mean calculated PPE-score of the 94 participants was 6.3 (95%-CI: 5.9-6.8), the median was 6.9 (interquartile range: 8-5 = 3). Firemen had the highest PPE-score of all three groups. They applied PPE significantly more frequently and completely than government workers (p = 0.006; Table 2). Both the PPE-device specific adherence ratio and the proportion of participants reporting having always been adherent to wearing the respective PPE-device were highest for gloves, second for protective boots and lowest for masks (Table 3). FFP3 (33/72) and multilayer surgical masks (29/72) were mostly applied among participants whom reported having always or sometimes used masks during collection of wild birds. No differences among the kind of applied masks regarding mean score of masks were found (p = 0.11).

**Table 2:** Mean PPE-score by occupation and difference in reference to fireman

	Mean PPE-score (95% CI)	Difference	p
**Fireman (n = 70)**	6.6 (6.1-7.2)	ref.	ref.
**Veterinarian (n = 5)**	5.9 (4.8-7.0)	0.7	0.12
**Government** **worker (n = 19)**	5.3 (4.4-6.1)	1.3	0.006

**Table 3:** Adherence ratio and proportion of adherent personnel for each PPE-device (n = 94)

	Maximum possible score for all participants	Actual score for all participants	Adherence ratio	Proportion of adherent personnel (always)
**Gloves**	188	166	88%	78%
**Protective boots**	94	77	82%	70%
**Protective clothing**	188	149	79%	63%
**Headwear**	94	68	73%	55%
**Protective goggles**	94	48	51%	37%
**Masks**	188	87	46%	19%

### Problems regarding PPE use

Any difficulties in adherence to recommended PPE use were reported by 24 of the 88 participants answering this question. The most commonly reported problems were short supply of PPE-devices (5/24) and mobility constraints (3/24). More specifically, 25/86 participants reported PPE-devices had interfered with their work, particularly protective goggles, masks and protective clothing. Regarding behaviour of personnel tasked with bird collection that potentially resulted in reduction of or gaps in the protective effect of PPE, 45% (41/92) participants reported using a mobile phone at least once during bird collection and 33% (30/92) reported driving an automobile while wearing protective clothing.

### Influenza vaccination status

Of 89 participants, 42 (47%) reported receipt of a seasonal influenza vaccination from July 2005 to February 2006. Altogether, 16/89 (18%) had received influenza vaccination for the season 2005/06 in 2005; 26/89 (29%) were vaccinated in February 2006 just prior to the collection of potentially infected birds.

### Acute respiratory symptoms during and after collecting birds

Of 90 participants, 7 (8%) reported symptoms of acute respiratory diseases during the period of bird collection or up to 5 days thereafter. Reported symptoms were cough (7/7), cold (5/7), headache (4/7), and muscle or limb pain (4/7). No participant reported fever. Thus, there were no reports that fulfilled the case definition of influenza-like illness. No differences among PPE-score (p = 0.33) and influenza vaccination status (p = 0.28) regarding acquiring acute respiratory symptoms were found.

### Serological analysis

Blood samples were provided by 78/97 (80%) participants. All serum samples were screened by PN assay at GNRCI; 5 sera were reactive against H5. Three of the 5 sera were tested by MN assay at GNRCI of which only one showed reduced viral replication. Retesting of the 5 reactive sera by MN assay at WHO-CC gave negative results. The 5 sera reactive in the PN assay were also tested by MN assay with human influenza A virus, and all showed very high antibody-titres against influenza A/H1N1 compared to A/H3N2. Information on influenza vaccination status was available for 4 of the 5 study participants whose sera were reactive in PN assay by GNRCI. All had received at least one seasonal influenza vaccination prior to the serological analysis (September 2005 to December 2006). However, no association was found between human influenza vaccination status and the PN assay result among all study participants (p = 0.13). An overview of information on occupation, PPE use, influenza vaccination status, respiratory symptoms, H5-titres, H1-titres and H3-titres of these 5 participants is presented in Table 4.

**Table 4:** Characteristics and serological titres of participants, whose sera were H5-reactive in PN assay at GNRCI

Occupation	PPE-Score	Influenza vaccination (09.2005-12.2006)	Respiratory symptoms	H5-titre (GNRCI)		H1-titre	H3-titre
				
				PN	MN	MN	MN
fireman	4	yes	no	1:208	1:240	5,120	< 1:80
fireman	6	yes	no	1:24	not tested	1,280	< 1:80
fireman	6	yes	no	1:68	not tested	320	< 1:80
fireman	8	no response	no	1:47	< 1:10	2,560	< 1:80
fireman	3	yes	no	1:33	< 1:10	1,280	< 1:80

## Discussion

To our knowledge, this is the first systematic assessment of adherence to recommended protective measures used by personnel tasked with bird collection during a large outbreak of HPAI A/H5N1 in wild birds.

The environmental conditions during wild bird collection differed considerably from culling of poultry during outbreaks of avian influenza. Study participants reported difficulties owing to a wet and cold environment during wild bird collection, and almost half the participants collected potentially infected birds that were still alive which resulted in a high risk of exposure. These environmental conditions were reported to be favourable for virus survival as the A/H5N1 viruses are more stable in wet and fresh feces of infected animals [15,16].

In contrast to other studies assessing adherence to recommended preventive measures during outbreaks of HPAI, we not only measured the proportion of PPE always used, but constructed a score to summarize both the completeness and frequency of PPE use simultaneously considering differences in the protective effect of PPE-devices. Studies conducted after the HPAI A/H7N7 outbreak in poultry in the Netherlands in 2003 showed a low self-reported adherence in the consistent use of masks and protective goggles among poultry farmers (6%, 1%) and cullers (25%, 13%) [17]. Based on the PPE score, our study showed that PPE adherence differed between occupational groups as well, and was highest in firemen who probably similar to cullers had more previous experience in the use of PPE owing to their occupation. However, this estimation requires confirmation by further investigations. Our study showed better adherence to using protective goggles among all personnel tasked with bird collection (37%). Compared with the results of the study after an A/H7N3 outbreak in poultry in Canada in 2004 (always use masks: 83%, gloves: 85% and protective goggles: 55%) the adherence in our study was poor (19%; 78%; 37%) [18]. However, the better result for adherence measured using the PPE-score method (46%, 88%, 51%), suggests an underestimation of the true adherence when restricting the analysis to the consistent use of PPE.

Interference of PPE with the task of wild bird collection was reported in particular for protective goggles and masks, in keeping with the lowest adherence for these two PPE-devices. The use of mobile phones during bird collection could have reduced the adherence of mask use as well. To our knowledge, other studies have not specifically addressed the question of gaps or barriers reducing adherence.

Since 2003, the German Committee for Biological Agents has recommended seasonal influenza vaccination for people exposed to A/H5N1 infected birds or poultry [6]. Even though seasonal vaccination does not protect against infection with avian influenza, it can potentially reduce opportunities for reassortment by avoiding the simultaneous infection of humans with avian and human influenza viruses. After an influenza vaccination, the development of an immune response takes about 2 weeks [19]. In our study, all participants had been offered seasonal influenza vaccination. However, 53% of those were unvaccinated. Among all participants, 29% had received a seasonal influenza vaccination only shortly before bird collection in February 2006 who may not have developed immunity against seasonal influenza during the first few days of bird collection. The proportion of study participants with a seasonal influenza vaccination prior to the outbreak (18%) in our investigation was similar to the proportion in a study carried out after an outbreak of A/H7N3 in Canada (21%) [18] and in a study after an outbreak of HPAI A/H5N1 in England (16%) [20]. However, this proportion is lower than influenza immunization coverage in the general population of Germany in the season 2005/06 (32.5%) [21].

In our study, differences were found between PN and MN results. Five sera reactive to A/H5N1 in PN assay could not be confirmed by MN assay. Discrepant results between serological assays have been shown for sera in the study in the Netherlands as well. A/H7N7-reactive sera initially tested by haemagglutination inhibition (HI) assay were all negative by an MN assay [22]. Therefore, the MN assay showed higher specificity than PN and HI assays. The lack of concordance of results between GNRCI and WHO-CC might be also explained by the use of different reference strains, even though both belong to clade 2.2.

A possible cross-reaction of A/H1N1 antibodies in high concentrations with the A/H5N1 reference virus was found by MN assay at GNRCI. It has been postulated that seasonal influenza vaccination may boost the anti-N1 response and therefore could lead to false-positive results against A/H5N1 virus as well because of cross-reactions between A/H1N1 and A/H5N1 [23]. As no association between human influenza vaccination status and PN assay result among study participants was found, it is unclear whether the cross-reaction between A/H5N1 and A/H1N1 could explain the reactivity by PN assay.

A limitation of our study is its conduct one year after the outbreak. Some study participants might have been unable to remember the details of their activities during the outbreak in 2006, which could reduce the validity of the study findings. Also the influenza antibody titres could decay over time [24], so the serological investigation might have failed to reveal seroconversion to H5 owing to the length of time elapsed since exposure.

Because soldiers of the German Federal Defence Force and professional firemen could not be included in this study, this investigation was based on participation of local personnel who performed initial response to the outbreak. As this study conducted on Ruegen was voluntary, not all individuals involved in bird collection were included. Participation among government workers and veterinarians was high at > 80%. Therefore, this study provides a good assessment for these two groups. The participation among firemen, however, was lower (55%) and might lead to selection bias as possibly firemen taking part in this study were more highly motivated and interested and used PPE more intensively than their non-responding colleagues. Therefore, the findings from this group could be less reliable. This could be another limitation of this study.

Evidence of the risk factors or protective effect of the protective measures could not be further analysed in our study because no A/H5N1-positive case or influenza-like illness was detected. Our findings should be completed by future studies under other operating conditions.

## Conclusion

As every human infection with avian influenza presents a chance for further adaptation of the virus and might lead to severe disease with a high case fatality, adherence to recommendations for the use of protective measures needs to be improved to reduce the risk of exposure to A/H5N1 infection.

Personnel with potential involvement in bird collection during wild bird outbreaks should be identified in advance and offered early and regular training in PPE use, particularly regarding masks and protective goggles use and the environmental conditions of wild bird collection. Only persons vaccinated against seasonal influenza should be admitted participating in bird collection.

Problems regarding PPE use and behavioural risk factors should be taken into account by the recommendations to avoid gaps in the use of PPE or reduction of its protective efficacy. Recommendations should also consider aspects of work organisation to prevent avoidable risks, e.g. concerning assignment of separate personnel for transport or communication.

Possible exposure to infected animals and adherence to recommendations should be assessed systematically in a timely manner. The potential risk for bird to human transmission - even if could not be quantified - during HPAI outbreaks among wild birds justifies early follow-up of exposed persons by means of serological testing using high-specificity assays.

## Competing interests

The authors declare that they have no competing interests.

## Authors' contributions

WC participated in the design and coordination of the study, data and blood collection, performed the statistical analysis and drafted the manuscript. BS participated in the design of the study and carried out the serological testing. UB participated in the design of the study, data and blood collection, assisted in the statistical analysis and helped to draft the manuscript. SB participated in the design and coordination of the study, data and blood collection and helped to draft the manuscript. ML participated in the design of the study. JH participated in the design and coordination of the study, data and blood collection. WH participated in the design and coordination of the study, data and blood collection, assisted in the statistical analysis, helped to draft the manuscript and made final revisions of the manuscript. All authors read and approved the final manuscript.

## Pre-publication history

The pre-publication history for this paper can be accessed here:

http://www.biomedcentral.com/1471-2334/9/170/prepub

## Supplementary Material

Additional file 1 **Questionnaire**. Questionnaire for personnel tasked with bird collection who participated in the study. Click here for file

Additional file 2 **Questionnaire**. English translation of the questionnaire for personnel tasked with bird collection who participated in the study. Click here for file
