# Supplementary material for: Protective measures and H5N1-seroprevalence among personnel tasked with bird collection during an outbreak of avian influenza A/H5N1 in wild birds, Ruegen, Germany, 2006
Source: BMC Infect Dis. 2009 Oct 18;9:170. doi: 10.1186/1471-2334-9-170 (PMC2767352; doi:10.1186/1471-2334-9-170)
Supplement: Additional file 1 — Questionnaire. Questionnaire for personnel tasked with bird collection who participated in the study. [file 1471-2334-9-170-S1.PDF]

Survey von Personen, die am Aufsammeln von Vögeln auf Rügen im Februar/März 2006 beteiligt waren

Fragebogen für Aufsammler

Datum der Befragung: ☐☐.☐☐.☐☐☐☐ (Datum angeben im Format TT.MM.JJJJ)

Persönliche Daten

1. Vorname, Name: ……………. ……………

2. Telefonnummer: ……………………………

3. Geschlecht: ❐₁ männlich ❐₂ weiblich

4. Geburtsdatum: ☐☐.☐☐☐☐ (Datum angeben im Format MM.JJJJ)

5. Gruppenzugehörigkeit:
- ❐₁ Freiwillige Feuerwehr
- ❐₂ Berufsfeuerwehr
- ❐₃ Mitarbeiter der Ordnungsämter/Kommunen
- ❐₄ Mitarbeiter der Veterinärämter
- ❐₅ Bundeswehr
- ❐₆ andere Berufsgruppe *(bitte angeben)*:………………………

6. Rauchen Sie?

❐₁ Ja ❐₂ Nein

↳ **Falls ja, wie viele Zigaretten rauchen Sie pro Tag?**

❐₁ weniger als 5 pro Tag ❐₂ 5 - 10 pro Tag ❐₃ 11-20 pro Tag ❐₄ mehr als 20 pro Tag

7. War(en) bei Ihnen vor Beginn der Aufsammeltätigkeit eine oder mehrere der folgenden Diagnosen gestellt worden
- ❐₁ Asthma
- ❐₂ Chronische Bronchitis
- ❐₃ Chronisch obstruktive Lungenkrankheit
- ❐₄ Herzinsuffizienz oder Herzschwäche
- ❐₅ Koronare Herzkrankheit oder Angina pectoris
- ❐₆ Herzinfarkt
- ❐₇ keine der angegebenen Diagnosen

Exposition

8. In welchen Zeitraum haben Sie die Vögel aufgesammelt? (mehrere Angaben möglich)

❐₁ 04.02.- 19.02.2006 ❐₂ 20.02-12.03.2006 ❐₃ anderer Zeitraum (*bitte angeben:* …………………)

9. Bitte füllen Sie die Tabelle aus, und kreuzen Sie zutreffende Angaben an.

Arbeitszeiten		**04.02.-19.02.2006**				**20.02.-12.03.2006**			
**Waren Sie in diesen Zeiträumen am Aufsammeln von Vögeln auf Rügen beteiligt?**		***Bitte die Anzahl der Arbeitstage mit Aufsammeltätigkeit in der jeweiligen Woche angeben:***<br>1. **Sa, 04.02. - So, 12.02.** ……..Tage<br>2. **Mo, 13.02. - So, 19.02.** ……..Tage				***Bitte die Anzahl der Arbeitstage mit Aufsammeltätigkeit in der jeweiligen Woche angeben:***<br>1. **Mo, 20.02. --So, 26.02.** ……..Tage<br>2. **Mo, 27.02. – So, 05.03.** ……...Tage<br>3. **Mo, 06.03. – So, 12.03.** ……..Tage			
Vogelspezies		**Schwan**	**Wildgans**	**Wildente**	**Un-bekannt**	**Schwan**	**Wildgans**	**Wildente**	**Un-bekannt**
**Welche Vogelspezies haben Sie aufgesammelt? Bitte schätzen Sie die <u>Anzahl pro Tag</u>.**									
**In welchen Regionen haben Sie die Vögel auf-gesammelt?** *(Bitte alle zutreffende ankreuzen)*	Zentral-Rügen (Mutland)								
	Halbinsel Jasmund								
	Wittow								
	Südost-Rügen (Granitz & Mönchgut)								
	West-Rügen								
	Südwest-Rügen								
	Süd-Rügen								
	Insel Hidden-see								
**Von wo haben Sie die Vögel auf-gesammelt?** *(Bitte alle zutreffende ankreuzen)*	auf dem Boden liegend								
	im Wasser oder Eis								
**Die von Ihnen auf-Gesammel-ten Vögel waren…** *(Bitte alle zutreffende ankreuzen)*	Fest-gefroren								
	nass								
	trocken								
**Waren Vögel darunter, die noch lebten?**		❒$_1$ Ja<br>❒$_2$ Nein	❒$_1$ Ja<br>❒$_2$ Nein	❒$_1$ Ja<br>❒$_2$ Nein	❒$_1$ Ja<br>❒$_2$ Nein	❒$_1$ Ja<br>❒$_2$ Nein	❒$_1$ Ja<br>❒$_2$ Nein	❒$_1$ Ja<br>❒$_2$ Nein	❒$_1$ Ja<br>❒$_2$ Nein
**Haben Sie beim Einpacken der Vögel mitgeholfen?**		❒$_1$ Ja<br>❒$_2$ Nein	❒$_1$ Ja<br>❒$_2$ Nein	❒$_1$ Ja<br>❒$_2$ Nein	❒$_1$ Ja<br>❒$_2$ Nein	❒$_1$ Ja<br>❒$_2$ Nein	❒$_1$ Ja<br>❒$_2$ Nein	❒$_1$ Ja<br>❒$_2$ Nein	❒$_1$ Ja<br>❒$_2$ Nein
**Hatten Sie bei einer anderen Tätigkeit Kontakt zu den aufgesammelten Vögeln?**		❒$_1$ Ja<br>❒$_2$ Nein	❒$_1$ Ja<br>❒$_2$ Nein	❒$_1$ Ja<br>❒$_2$ Nein	❒$_1$ Ja<br>❒$_2$ Nein	❒$_1$ Ja<br>❒$_2$ Nein	❒$_1$ Ja<br>❒$_2$ Nein	❒$_1$ Ja<br>❒$_2$ Nein	❒$_1$ Ja<br>❒$_2$ Nein

Schutzmaßnahmen

10. Waren Sie vor dem Beginn der ersten Aufsammeltätigkeit arbeitsmedizinisch untersucht (G26)?

❒1 Ja *(Bitte das Untersuchungsdatum angeben)*: □□.□□.□□□□

❒2 Nein

❒3 weiß nicht

11. Sind Sie vor dem Beginn der ersten Aufsammeltätigkeit über den korrekten Umgang mit der Schutzausrüstung aufgeklärt worden?

❒1 Ja ❒2 Nein ❒3 weiß nicht

↳ **Falls ja:**

❒1 durch das Gesundheitsamt

❒2 durch eine andere Institution

↳ **Falls nein, welche weiteren Informationen hätten Sie noch gebraucht?** ………………………………………………………...

…………………………………………………………………………..

12. Bitte geben Sie die von Ihnen getroffenen Schutzmaßnahmen an.

	04.02.-19.02.2006				20.02.-12.03.2006			
	Ja, immer	Teilweise	Nein	Weiß nicht	Ja, immer	Teilweise	Nein	Weiß nicht
**Körperbedeckende Arbeitsschutzkleidung**								
**Kopfbedeckung**								
**Desinfizierbare Stiefel**								
**Desinfizierbare Schutzhandschuhe**								
**Atemschutz / Maske**								
(Falls ja oder teilweise, bitte die von Ihnen verwendeten ankreuzen):	❒1 mehrlagige dichtschließende OP-Maske<br>❒2 FFP 1<br>❒3 FFP 2<br>❒4 FFP 3<br>❒5 Atemschutzgerät<br>❒6 weiß nicht				❒1 mehrlagige dichtschließende OP-Maske<br>❒2 FFP 1<br>❒3 FFP 2<br>❒4 FFP 3<br>❒5 Atemschutzgerät<br>❒6 weiß nicht			
**Augenschutz**								
**Händedesinfektion vor Beginn von Pausen**								
**Händedesinfektion nach Beendigung der Aufsammeltätigkeit**								
**Getrennte Aufbewahrung der Straßenkleidung von der Arbeitskleidung und der persönlichen Schutzausrüstung**								

13. Gab es während des Aufsammelns Schwierigkeiten, die empfohlenen Schutzmaßnahmen einzuhalten?

❒1 Ja ❒2 Nein ❒3 weiß nicht

➯ **Falls ja, bitte die Schwierigkeiten angeben.**

………………………………………………………………………………………………

14. Gab es Schutzmaßnahmen, die Sie in ihrer Tätigkeit behindert haben?

❒1 Ja ❒2 Nein ❒3 weiß nicht

➯ **Falls ja, bitte geben Sie die Schutzmaßnahmen und die entsprechenden Gründe an.**

❒1 Körperbedeckende Arbeitsschutzkleidung (*Bitte Gründe angeben):* …………………………….

❒2 Kopfbedeckung (*Bitte Gründe angeben):* ………………………………………………………….

❒3 Desinfizierbare Stiefel (*Bitte Gründe angeben):* ……………………………………………….

❒4 Desinfizierbare Schutzhandschuhe (*Bitte Gründe angeben):* …………………………………….

❒5 Atemschutz / Maske (*Bitte Gründe angeben):* ……………………………………………………..

❒6 Augenschutz (*Bitte Gründe angeben):* ……………………………………………………………..

15. Sind Sie mit Arbeitsschutzkleidung mit einem PKW oder anderem Transportmittel gefahren?

❒1 Ja ❒2 Nein ❒3 weiß nicht

16. Haben Sie während des Aufsammelns mindestens 1-mal ein Handy benutzt?

❒1 Ja ❒2 Nein ❒3 weiß nicht

17. Wurde Ihnen empfohlen, das Medikament Tamiflu® (Oseltamivir) einzunehmen?

❒1 Ja ❒2 Nein ❒3 weiß nicht

18. Haben Sie das Medikament Tamiflu® (Oseltamivir) eingenommen?

❒1 Ja ❒2 Nein ❒3 weiß nicht

➯ **Falls ja, wann haben Sie begonnen, Tamiflu® einzunehmen?**

❒1 vor der Aufsammeltätigkeit

❒2 nachdem ich schon mit der Aufsammeltätigkeit begonnen hatte

❒3 nach Beendigung der Aufsammeltätigkeit

❒4 weiß nicht

Über welchem Zeitraum hatten Sie Tamiflu® eingenommen? Bitte berechnen Sie den Zeitraum vom ersten Tag bis zum letzten Tag der Einnahme, einschließlich Unterbrechungen, bitte geben Sie die Anzahl der Tage an (ggf. bitte schätzen).

……………... **Tage**

Hatten Sie die Einnahme von Tamiflu® unterbrochen / vorzeitig beendet?

❒1 Ja ❒2 Nein

➯ **Falls ja, was waren die Gründe?** (<u>mehrere Angaben</u> möglich)

❒1 vergessen ❒2 Übelkeit ❒3 Durchfall ❒4 andere (*Bitte angeben):* …………….

Wie viele Tage hatten Sie insgesamt in dem von Ihnen angegebenen Zeitraum keine Kapsel eingenommen? ……………... **Tage**

19. Bitte geben Sie alle Grippeschutzimpfungsdaten vom Juli 2005 bis jetzt an.

(Datum angeben im Format: MM.JJJJ)

1. ☐☐.☐☐☐☐ **2.** ☐☐.☐☐☐☐ **3.** ☐☐.☐☐☐☐

❐$_{1}$ Ich hatte seit Juli 2005 keine Grippeschutzimpfung

❐$_{2}$ weiß nicht

Atemwegserkrankung

20. Hatten Sie im Rahmen Ihrer Aufsammeltätigkeit eine Atemwegserkrankung?

❐$_{1}$ Ja ❐$_{2}$ Nein ❐$_{3}$ weiß nicht

➘ **Falls ja, wann hat die Atemwegserkrankung begonnen?**

❐$_{1}$ in den 5 Tagen vor Beginn der Aufsammeltätigkeit

❐$_{2}$ während dem Aufsammelzeitraum

❐$_{3}$ bis 5 Tage nach Ende des letzten Aufsammelns

❐$_{4}$ anderer Zeitraum

❐$_{5}$ weiß nicht

Welche Symptome hatten Sie? (mehrere Angaben möglich)

❐$_{1}$ Fieber ❐$_{2}$ Husten ❐$_{3}$ Kopfschmerzen ❐$_{4}$ Muskel- bzw. Gliederschmerzen ❐$_{5}$ Schüttelfrost

❐$_{6}$ Schnupfen ❐$_{7}$ Atemnot ❐$_{8}$ andere *(Bitte angeben)*: …………………………….. ❐$_{9}$ weiß nicht

Wie rasch begannen Ihre Symptome?

❐$_{1}$ plötzlich ❐$_{2}$ allmählich ❐$_{3}$ weiß nicht

Waren Sie wegen dieser Erkrankung zum Arzt gegangen?

❐$_{1}$ Ja ❐$_{2}$ Nein ❐$_{3}$ weiß nicht

Welche Diagnose hat Ihr Arzt (Ihre Ärztin) festgestellt?

(Bitte angeben): ……………………………

Wurden Sie wegen dieser Erkrankung ins Krankenhaus eingewiesen?

❐$_{1}$ Ja ❐$_{2}$ Nein ❐$_{3}$ weiß nicht

Vielen Dank für Ihre Antwort!

Bitte schicken Sie den ausgefüllten Fragebogen mit dem beiliegenden frankierten Rückumschlag zurück an:

Robert Koch-Institut
Abteilung für Infektionsepidemiologie
Frau Cai
Postfach 65 02 61
13302 Berlin
