# Supplementary material for: Protective measures and H5N1-seroprevalence among personnel tasked with bird collection during an outbreak of avian influenza A/H5N1 in wild birds, Ruegen, Germany, 2006
Source: BMC Infect Dis. 2009 Oct 18;9:170. doi: 10.1186/1471-2334-9-170 (PMC2767352; doi:10.1186/1471-2334-9-170)
Supplement: Additional file 2 — Questionnaire. English translation of the questionnaire for personnel tasked with bird collection who participated in the study. [file 1471-2334-9-170-S2.PDF]

State Office of
Health and Social Affairs
Mecklenburg-Western Pomeriania

ROBERT KOCH INSTITUT

Survey of personnel tasked with bird collection on the island of Ruegen from February to March 2006

Questionnaire for personnel tasked with bird collection

Date of interview: ☐☐.☐☐.☐☐☐☐ (date in DD.MM.YYYY)

Personal data

1. First name, name: ……………. ……………

2. Telephone number: ……………………………

3. Gender: ❒1 male ❒2 female

4. Date of birth: ☐☐.☐☐☐☐ (date in MM.YYYY)

5. Group membership:
❒1 firemen of auxiliary fire brigade
❒2 professional firemen
❒3 staff member of the municipal public order authority
❒4 veterinary office staff member
❒5 Federal Armed Forces
❒6 other profession *(Please, indicate)*:…………………

6. Do you smoke?
❒1 Yes ❒2 No
➭ **If yes, how many cigarettes do you smoke per day?**
❒1 less than 5 per day ❒2 5 - 10 per day ❒3 11-20 per day ❒4 more than 20 per day

7. Were you diagnosed for any of the following diseases prior to your employment as personnel tasked with bird collection?
❒1 asthma
❒2 chronic bronchitis
❒3 chronic obstructive pulmonary disease
❒4 congestive heart failure or chronic cardiac insufficiency
❒5 coronary heart disease or angina pectoris
❒6 myocardial infarction
❒7 none of the diseases mentioned above

State Office of
Health and Social Affairs
Mecklenburg-Western Pomeriania

ROBERT KOCH INSTITUT

Exposition

8. During which time period did you collect birds? (<u>multiple answers</u> possible)

❐1 04.02.- 19.02.2006 ❐2 20.02.-12.03.2006 ❐3 another time period (*please, indicate*…………………)

9. Please, fill out the table and mark the appropriate answers with a cross.

Time period of work		**04.02.-19.02.2006**				**20.02.-12.03.2006**			
**Did you participate in collecting birds on the island of Ruegen in the indicated time period?**		***Please, indicate the number of working days, that you were tasked with bird collection, in each particular week:***<br><br>**Sat 04.02. - Sun 12.02.** …….days<br>**Mon 13.02. - Sun 19.02.** …….days				***Please, indicate the number of working days, that you were tasked with bird collection, in each particular week:***<br><br>1. **Mon 20.02. --Sun 26.02.** …….days<br>2. **Mon 27.02. – Sun 05.03.** ……...days<br>3. **Mon 06.03. – Sun 12.03.** …….days			
Bird species		**swan**	**wild goose**	**mallard**	**unknown**	**swan**	**wild goose**	**mallard**	**unknown**
**Which bird species did you collect? Please, estimate the number of collected birds per day.**									
**In which area did you collect the birds?** *(Please, mark appropriate answers)*	Central Ruegen (Mutland)								
	Peninsula Jasmund								
	Wittow								
	South-eastern Ruegen (Granitz & Moenchgut)								
	Western Ruegen								
	South-western Ruegen								
	Southern Ruegen								
	Island of Hidden-see								
**Wherefrom did you collect the birds?** *(Please, mark appropriate answers)*	lying on the ground								
	in water or ice								
**The birds you collected were …** *Please, mark appropriate answers)*	frozen								
	wet								
	dry								
**Were there birds included, which were still alive?**		❐1 Yes<br>❐2 No	❐1 Yes<br>❐2 No	❐1 Yes<br>❐2 No	❐1 Yes<br>❐2 No	❐1 Yes<br>❐2 No	❐1 Yes<br>❐2 No	❐1 Yes<br>❐2 No	❐1 Yes<br>❐2 No
**Did you help packaging the birds?**		❐1 Yes<br>❐2 No	❐1 Yes<br>❐2 No	❐1 Yes<br>❐2 No	❐1 Yes<br>❐2 No	❐1 Yes<br>❐2 No	❐1 Yes<br>❐2 No	❐1 Yes<br>❐2 No	❐1 Yes<br>❐2 No
**Were you exposed to the collected birds while performing another task?**		❐1 Yes<br>❐2 No	❐1 Yes<br>❐2 No	❐1 Yes<br>❐2 No	❐1 Yes<br>❐2 No	❐1 Yes<br>❐2 No	❐1 Yes<br>❐2 No	❐1 Yes<br>❐2 No	❐1 Yes<br>❐2 No

Precautions/protective measures

10. Did you receive an occupational health checkup prior to your first assignment as personnel tasked with bird collection (G26)?

❐1 Yes *(Please, indicate the date of the checkup)*: ☐☐.☐☐.☐☐☐☐
❐2 No
❐3 don't know

11. Were you instructed in the correct handling of the personal protective equipment prior to your first assignment as personnel tasked with bird collection?

❐1 Yes
↳ **If Yes:**
❐1 by health authorities
…………………………
❐2 by another institution

❐2 No
↳ **If No, which further information would you have appreciated?**
………………………………………………

❐3 don't know

12. Please, indicate precautions that you've followed.

	04.02.-19.02.2006				20.02.-12.03.2006			
	Yes, always	sometimes	No	don't know	Yes, always	some-times	No	don't know
**Protective clothing**								
**Headwear**								
**Protective boots**								
**Protective gloves**								
**Respirator / masks**								
(If Yes or sometimes, please mark the type you used):	❐1 multilayer firmly sealed surgical mask<br>❐2 FFP 1<br>❐3 FFP 2<br>❐4 FFP 3<br>❐5 respirator<br>❐6 don't know				❐1 multilayer firmly sealed surgical mask<br>❐2 FFP 1<br>❐3 FFP 2<br>❐4 FFP 3<br>❐5 respirator<br>❐6 don't know			
**Protective goggles**								
**Disinfection of hands prior to breaks**								
**Disinfection of hands after finishing the task of bird collection**								
**Separate storage of street clothes from protective clothing and personal protective equipment**								

13. Did you encounter difficulties following the recommended protective measures while collecting the birds?

❒1 Yes ❒2 No ❒3 don't know

↳ **If Yes, please specify the difficulties.**

……………………………………………………………………………………

14. Were there protective measures that you felt constrained your work?

❒1 Yes ❒2 No ❒3 don't know

↳ **If Yes, please indicate the protective device and the reasons, respectively.**

❒1 **Protective clothing** (*Please, indicate reasons):*
……………………………………………………………..
❒2 **Headwear** *(Please, indicate reasons):*
……………………………………………………………
❒3 **Protective boots** *(Please, indicate reasons):*
……………………………………………………………
❒4 **Protective gloves** *(Please, indicate reasons):*
……………………………………………………………..
❒5 **Respirator / masks** (*Please, indicate reasons):*
……………………………………………………………
❒6 **Protective goggles** (*Please, indicate reasons):*
……………………………………………………………

15. Did you drive a car or use other transportation wearing protective clothing?

❒1 Yes ❒2 No ❒3 don't know

16. Did you use your cell phone at least once while collecting birds?

❒1 Yes ❒2 No ❒3 don't know

17. Were you advised to take the antiviral medication Tamiflu® (Oseltamivir)?

❒1 Yes ❒2 No ❒3 don't know

18. Did you take the antiviral medication Tamiflu® (Oseltamivir)?

❒1 Yes ❒2 No ❒3 don't know

↳ **If Yes, when did you start taking Tamiflu®?**

❒1 prior to the work as personnel tasked with bird collection
❒2 after starting the work as personnel tasked with bird collection
❒3 after finishing the work as personnel tasked with bird collection
❒4 don't know

19. How long did you take Tamiflu®? Please, calculate the period from the first to the last day of taking it, including interruptions, please indicate the number of days (if necessary estimate). …………….. days

20. Did you interrupt or finish the taking the antiviral medication Tamiflu® earlier than recommended?

❒1 Yes ❒2 No

↳ **If Yes, for what reasons?** (multiple answers possible)

❒1 forgot ❒2 Dausea ❒3 Diarrhea ❒4 Others *(Please, indicate):* …………….

For how many days in total did you not take a capsule of antiviral medication during the indicated period? …………….. days

21. Please, indicate all dates of influenza vaccination between July 2005 and today.
(date in: MM.YYYY)

1. ☐☐.☐☐☐☐ **2.** ☐☐.☐☐☐☐ **3.** ☐☐.☐☐☐☐

❒1 I have not received any influenza vaccination since July 2005
❒2 don't know

Respiratory infection

22. Did you suffer from respiratory infections in the course of your work as personnel tasked with bird collection?

❒1 Yes ❒2 No ❒3 don't know

↳ **If Yes, when did the symptoms start?**

❒1 within 5 days before collecting birds
❒2 during the task of collecting of birds
❒3 within 5 days after finishing the last bird collection
❒4 another period
❒5 don't know

Which symptoms did you have? (multiple answers possible)

❒1 fever ❒2 cough ❒3 headache ❒4 muscle / limb aches
❒5 chills ❒6 cold ❒7 shortness of breath ❒8 others *(please, indicate)*:……………………
❒9 don't know

How fast did your symptoms start?

❒1 suddenly ❒2 gradually ❒3 don't know

Did you visit a doctor because of this disease?

❒1 Yes ❒2 No ❒3 don't know

What was your doctor's diagnosis?
(Please, indicate): ……………………………

Were you hospitalized because of this disease?

❒1 Yes ❒2 No ❒3 don't know

Thank you very much for answering!

Please, return the filled in form using the enclosed stamped envelope to:

Robert Koch Institute
Department of Infectious Disease Epidemiology
